# Supplementary material for: Comparative Structural and Compositional Analyses of Cow, Buffalo, Goat and Sheep Cream
Source: Foods. 2021 Nov 1;10(11):2643. doi: 10.3390/foods10112643 (PMC8618205; doi:10.3390/foods10112643)
Supplement: Supplementary file 1 [file foods-10-02643-s001.zip › foods-1402550 supp.pdf]

Supplementary material

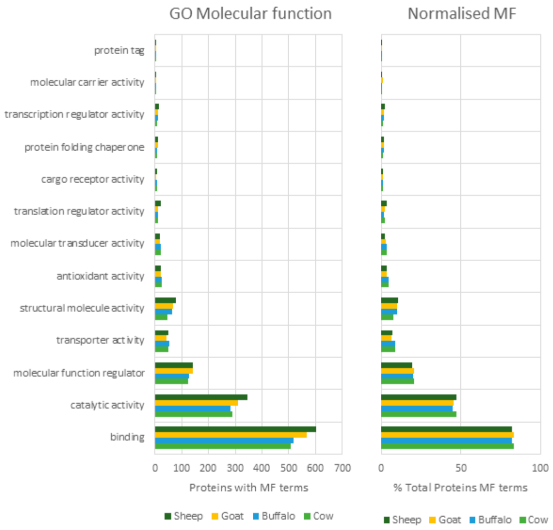

Figure S1. Summary of MF terms of protein groups from analysis of cream from 4 species, with absolute numbers (**left**) and normalised to total number of annotated proteins (**right**).

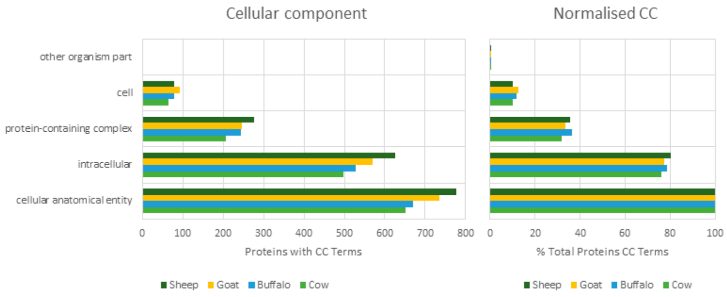

Figure S2. Summary of CC terms of protein groups from analysis of cream from 4 species, with absolute numbers (**left**) and normalised to total number of annotated proteins (**right**).
